# Effectiveness of peer-learning assisted primary school students educating the rural community on insecticide-treated nets utilization in Jimma-zone Ethiopia

**DOI:** 10.1186/s12936-020-03401-7

**Published:** 2020-09-11

**Authors:** Yohannes Kebede, Lakew Abebe, Guda Alemayehu, Morankar Sudhakar, Zewdie Birhanu

**Affiliations:** 1 Department of Health, Behavior, and Society, Jimma University, Jimma, Ethiopia; 2 President’s Malaria Initiative, United States Agency for International Development, Addis Ababa, Ethiopia

**Keywords:** Schoolchildren change agents, School-based health education, ITN utilization, Malaria elimination, Jimma-Zone, Ethiopia

## Abstract

**Background:**

Making insecticide-treated nets (ITNs) utilization a social norm would support the global goal of malaria eradication and Ethiopian national aim of its elimination by 2030. Jimma zone is one of the endemic settings in Ethiopia. This study aimed to report effects of malaria education, delivered by students, on community behaviours; particularly ITNs.

**Methods:**

In pre-posttest, cross-sectional household surveys conducted in rural villages of 5 districts in Jimma Zone, Ethiopia, 762 households were sampled. The intervention engaged students from primary schools in participatory peer education within small groups, followed by exposing parents with malaria messages aimed at influencing perceptions and practices. The data were analysed using SPSS version 20.0. Proportions/means differences were computed to compare changes in exposure, knowledge, perceptions, and practices using 95% CI at p < 0.05. Regression analyses were conducted to assess exposures to school-based education, content intensity, perception, and access related predictors of ITN utilization over the intervention periods.

**Results:**

Over the intervention periods, the findings showed significant improvement in exposure to and content intensity of malaria messages delivered by students, effect size (ES) = 44.5% and 19.3%, p < 0.001, respectively. ITN utilization (ES = 25.8%), and the reported behaviour of giving ITN priority to children under 5 years old and pregnant women increased by ES = 16.3% and  24.8%, respectively. The exposure status or content intensity of malaria education, in turn, significantly improved comprehensive knowledge about malaria (β = +1.82), misconceptions about causes (β = − 11.46), awareness of caring for ITN (β = +24.79), identifying ITN as effective preventive methods (OR = 1.93), attitude towards ITN (β = +0.20), perceived efficacy of ITN (OR = 1.04), acceptance of ITN as a means to control the danger of malaria (β = +8.08%), and ITN utilization (OR = 1.85). Nonetheless, perceived threat (β = − 0.19) significantly negatively correlated with exposure to students’ messages. Socio-demography, access, exposures to messages, and parental perception that students were good reminders predicted ITN utilization over the intervention periods with some changing patterns.

**Conclusions:**

Exposing the community to malaria education through students effectively supports behaviour change, particularly ITN usage, to be more positive towards desired malaria control practices. A school-based strategy is recommended to the national effort to combat malaria.

## Background

Despite significant reduction, malaria remains a public health challenge that deems global attention toward elimination and supports. Sub-Saharan Africa and India carried almost 85% of the global malaria burden [1–3]. A global malaria programme (GMP) aims to achieve elimination by 2035 and global eradication by 2040–2050 [4–6]. Ethiopia has been implementing national malaria strategic plans (NMSPs) phase III, aiming to meet the ambitious goal of eliminating malaria by 2030 [7–12]. The Ethiopian National Malaria Indicator Survey (ENMIS) 2015 revealed that 65% of districts in the country were malarious, and 53% had risk of moderate to high transmission. Jimma zone is one of the endemic settings [13–16]. The global malaria control strategy has been providing the support required to enhance the move towards malaria elimination [2–5]. Insecticide treated-nets (ITNs), indoor residual spray (IRS), accurate diagnosis, and prompt treatment by artemisinin-based combination therapy (ACT) are some of the resources [3–6]. In Ethiopia, across the NMSP phases (I-III), malaria prevention and control services have intensified, including the wide distribution of ITNs, and social and behaviour change communication (SBCC) strategies [13–18].

Utilization of ITNs has been one of the front-line measures of malaria prevention and core mosquito vector control methods as specified by World Health Organization (WHO) frame work of malaria elimination, Roll Back Malaria (RBM)/SBCC indicators, and Ethiopian NMSPs [12, 15, 17–22]. To address the frontline behaviours, the RBM-SBCC indicators identified helpful tools as community exposure to messages via different strategies, knowledge, risk perceptions, self-efficacy, social norms, and attitudes or perceived response efficacies. Ethiopian NMSPs delineate these tools to support positive behavior changes, including the use of ITNs, towards malaria elimination [7–12].

A school-based SBCC is an emerging and effective approach to promote public health. SBCC is an evidence-based approach, a theory supported and context-specific strategy that makes use of locally available opportunities in a given social or community setting [19–26]. Evidence indicates that SBCC resulted in improving malaria prevention and control through shifts in norms, knowledge, and particularly ITN utilization in different contexts [26–35]. Its effectiveness is much stronger when supported by access to resources (e.g. ITNs) [36–41].

Multiple school-based educations and school health initiatives have encompassed strategies aimed at improving capacity, knowledge, and decision-making skills to help promote health and prevent diseases among schoolchildren, their families, and beyond [42–45]. Previous school-based educations in different countries indicated several adaptable experiences. The first key lesson was that an objective-based and manual-supported provision of malaria education in schools significantly improved students’ awareness, health status and reduced school absenteeism. Secondly, participatory peer education among students resulted in enhanced students’ knowledge, students as effective health messengers and change agents, and efficient means to reach out to community with sufficient knowledge and practices (e.g., in Uganda, Ghana, Kenya, and Thailand [24, 25, 27, 28, 31, 44, 45]. School students were perceived to play a pivotal role in keeping the health of their families and communities.

The current study hypothesized that empowering students in primary schools on malaria prevention and control through training, communication material supports, and participatory peer discussion would be an effective strategy to reach out to the community with messages, knowledge, perceptions, and improve practices; ITN utilization.

## Methods

### Study design and settings

The study used pre-posttest, cross-sectional household surveys in 20 rural villages (*gandas*) in 5 districts in Jimma zone: Limmu-Kossa, Botor-Tolay, Gera, Shebe-Sombo, and Nono-Benja. The baseline survey was conducted in October 2017. After the baseline survey completed, the school-based and peer-learning assisted educational intervention was launched in January 2018. Throughout the intervention period, community members received malaria education through trained school students. Students were supported by focal-teachers at schools and local project coordinators. The end-line survey was conducted in March 2019.

### Population and sampling

Sample sizes for the baseline and endline surveys were determined using two population proportion formula [(n = (Z_α/2_ + Z_β_)^2^ * (P_1_(1 − P_1_) + P_2_(1 − P_2_))/(P_1_ − P_2_)^2^], where Z_α/2_ is the critical value of the normal distribution at α/2, at a confidence level of 95%, which gives a critical value of 1.96), Z_β_ is the critical value of the normal distribution at β (for a power of 80%, β is 0.2, and the critical value is 0.84), and to detect a minimum difference (P_1_ − P_2_) of 7.5% between the two proportions (baseline and endline) in ITN usage, where P_1_ = 38.0% was taken from a previous study in similar districts. The calculation yielded a total sample of 762 households (HHs) for each survey. To recruit respondents, a multi-stage sampling method was used. First, based on the probability proportional to the size of the total population and HHs, a sample size was allocated to each study district, *gandas* and 3 sub-divisions (*zoni*) of the *gandas*. The sampling frames of the HHs used registration books located in the respective *gandas*. The final sample HHs were selected using computer-generated random numbers and the selected HHs were traced through local guides. The status of sleeping under ITN was assessed for every HH’s member.

### School-based community education intervention

The intervention used empowered students in primary schools as messengers teaching parents or neighbors as a means to improve HH’s exposure to malaria messages, perceptions and practices. It consisted of engaging students in primary schools (5-8 grades) using students’ network (so called 1-to-5) who received training about malaria prevention, and participated in peer-to-peer discussion every other week. The details of the intervention content and procedure was presented in separate article [46].

The students educate the parents, and followed by trained focal teachers and the project’s local coordinators. Schools report their activities monthly. Review meetings are conducted every 3 months in every district to address challenges and share experiences. Improved access to resources (ITNs) that support behaviours are executed in collaboration with the health offices.

### Theoretical background of the intervention

The current study was based on a mix of communication and behaviour change theories. The conceptual orientation about exposure to message leading to change in perceptions and actions was derived from the extended parallel process model-EPPM [47]. According to EPPM, individual risk appraisal leads to protection motivation if the messages to which they are exposed promote credible, accessible, feasible, and usable risk aversion strategies. Otherwise, it could result in a defensive response. Perceived susceptibility and severity together build a perceived threat of malaria. Self-efficacy to implement effective responses (e.g. ITNs) make perceived efficacy of engagement in recommended actions. The response efficacy is interchangeably used with attitude (attitude was used in this article). The excess presence of perceived efficacy preventive measures compared to the perceived threat of malaria leads to positive evaluation and acceptance of the advised actions. Acceptance would later lead to the desired behaviour, uptake of ITNs in this case. According to the diffusion of innovation theory, any idea or strategy perceived as new will diffuse through organizations, existing structures and social systems by using credible change agents [48]. Schools as organizations and students as change agents are supposed to disseminate malaria messages to the rest of the community. To enhance the credibility of students, in addition to organized peer education, a promotion was carried out on school family days opening programmes.

### Instrument and measurements

Standard tools were adopted from RBM SBCC indicators, EPPM constructs, and other similar studies [3–5, 18–21, 47]. The questionnaires consisted of several parts. Part I: socio-demographic characteristics (age, gender, education, occupation, marital status, family size, etc.). Part II: respondents’ knowledge about malaria and its control measures through ‘yes’ or ‘no’ formats. Part III: perceived efficacy (attitude and self-efficacy); attitude; 16 items [8 behavioural beliefs and 8 values], and self-efficacy; 7 items. Part IV: Perceived threat: perceived susceptibility (7 items), and perceived severity (5 items). Part III and IV were measured using a three-point Likert scale. Part V: respondents and household members’ malaria prevention practices: mainly, ownership and access, and utilization of ITN. Part VI: exposure and recall of malaria messages delivered by students. Part VII: Parental perceived engagement roles of students.

### Operational definitions

#### Exposure

A multi-dimensional recall of malaria messages: reach, repetition, and content intensity. Exposure status (reach) referred to recall about exposure to students’ education over the previous 6 months. Content intensity is the proportion of person-to-all specific messages to which a respondent was exposed over the previous 6 months. Repetition is the proportion of person-to-all message sources to which a respondent was exposed over the previous 6 months.

#### Comprehensive knowledge

Summed-up from correct answers given to 9 knowledge items about malaria: signs (3), cause (1), prevention measures (3), and high-risk groups (2).

#### Overall attitude

The first 8 behavioural beliefs about the outcome of engaging in malaria control measures were separately weighted by their respective evaluation of effectiveness. The weighted items were summed up. The overall score ranged from 8 to 72. To make the scale comparable with other psychological constructs (e.g., self-efficacy, perceived susceptibility, severity), the overall score standardized to percentages by multiplying it by 100 and dividing by the maximum score. The standardized score referred to the percentage of favorable attitude.

#### Attitude towards ITNs

The weighted sum of 4 beliefs about outcome of using ITN weighted by how effective the outcome was to prevent malaria. The score of the scale ranged from 4 to 36.

#### Perceived threat

First, perceived susceptibility and severity items summed-up from their corresponding items, then, the scores standardized to percentage of perceived threat.

#### Self-efficacy

The summation of items was about self-confidence to engage in preventive measures as recommended towards the prevention and control of malaria.

#### Perceived efficacy

How efficiently people engaged in actions that they believed to be effective in managing the danger of malaria was summed up from standardized attitude and self-efficacy, and adjusted to percentage. The perceived efficacy of ITN was calculated from items about ITN.

#### ITN message acceptance

Overall messages about threats of malaria and the effectiveness of measures to which respondents were exposed initiated feelings with two alternatives of message acceptance. Alternative 1: excess perceived threat compared to perceived efficacy messages labeled respondents in ‘fear control zone’. Alternative 2: excess perceived efficacy compared to perceived threat messages that labeled respondents in ‘danger control’. According to the extended parallel process model [47], people in fear control become reluctant to engage in malaria preventive actions even in the presence of resources (ITN), while people in danger control strive towards the uptake of preventive actions. Acceptance of ITN was calculated from ITN items.

#### Perceived engagement of students

Post-exposure community’s perspective of students’ perceived role as malaria educators.

### Data collection

Data were collected through face-to-face interviewer-administered methods at HH level. Experienced enumerators participated in collecting data. Three-day training was given to enumerators and supervisors about the purpose of the study, instruments and data collection during both surveys. The data collection process was closely supervised by research teams. The questionnaires were checked for completeness and consistency daily. The supervisors addressed the questions of overseeing investigators in their daily audits.

### Data analysis

The data were analysed using SPSS version 20.0. To describe the data, statistics such as frequency, proportions, mean, and standard deviations were used. We analyzed the data based on operational definitions were followed during the analysis. ITN utilization reported based on every HH member’s status of sleeping under it during the last nights of the surveys. Bivariate analysis was performed (using independent sample t-test, and Chi-square) to compare changes in standardized scores or percentages of exposure, comprehensive knowledge, attitude, perceptions, and practices between baseline and endline surveys. These key variables were compared based on exposure status and content intensity of messages delivered by students using logistic or linear regression to assess changes that were due to the school-based intervention. The changes were labeled as effect sizes (ES). A 95% confidence interval and < 0.05 level of significance were used to declare statistically significant differences. The practice of ITN utilization that changed over time and based exposure status or content intensity was further analysed for predictors. Percentage changes in the magnitude of odds ratios over the intervention periods were referred to assess patterns of the predictors’ modifications based on socio-demographic, ownership-access of ITN, perceptions, and community’s perceived roles of the students.

### Ethical considerations

The study was reviewed and approved by the Jimma University Institutional Review Board (IRB). Official permission letters to undertake the study were obtained from the concerned bodies at all levels. Informed written consent was obtained from all study participants. The data and consent forms were deposited in a private and confidential cabinet.

## Results

### Background characteristics

Table 1 presents the details of the socio-demographic characteristics of the respondents. A total of 1521 HHs participated in the surveys; 762 baseline and 759 endline. Overall, 7785 people lived in sampled HHs. A majority of respondents were married (87%), Oromo (77%), Muslim (58.5%), and could not read or write (47.4%).

**Table 1:** Socio-demographic characteristics of respondents in rural settings of Jimma Zone, Ethiopia, 2017–2019 (baseline, n = 762; end line, n = 759)

Socio-demographic characteristics	Repeated surveys					
	Baseline		End-line		Combined	
	No	%	No	%	No	%
Districts						
Botor-Tolay [HH = 239]	699	17.4	606	16.1	1305	16.8
Nono-Benja [HH = 248]	704	17.5	651	17.3	1355	17.4
Limmu-Kossa [HH = 264]	589	14.6	615	16.4	1204	15.5
Gera [HH = 333]	849	21.1	759	20.2	1608	20.7
Shebe-Sombo [HH = 437]	1184	29.4	1129	30.0	2313	29.7
Total [HH = 1521]	4025	100	3760	100	7785	100
Sex of respondent						
Male	378	49.8	439	57.9	817	53.7
Female	384	50.2	320	42.1	704	46.3
Marital status						
Married	676	89.2	642	85.0	1318	87.1
Divorced	26	3.4	31	4.1	57	3.8
Widowed	41	5.4	29	3.8	70	4.6
Others	15	2.0	53	7.2	68	4.5
Education						
Cannot read or write	402	52.8	313	41.2	715	47.0
Read & write	81	10.6	80	10.6	161	10.6
Primary school	216	28.3	251	33.1	467	30.7
Secondary school	60	7.9	106	14.0	166	10.9
College and above	3	0.4	5	1.1	8	0.5
Religion						
Muslim	465	61.0	423	55.9	888	58.5
Orthodox	194	25.5	230	30.4	424	27.9
Protestant	103	13.5	104	13.7	207	13.6
Ethnicity						
Oromo	593	77.8	576	76.0	1169	76.9
Amhara	122	16.0	138	18.2	260	17.1
Others	45	5.9	54	7.0	99	6.5
Proportions of						
HHs with school child	535	70.3	458	60.4	986	65.2

### Community perceptions of students’ roles as malaria educators

Table 2 presents a key summary of a post-intervention community perspective on students as malaria educators and change agents. Students were believed on as credible messengers (64.3%), reminders mainly for every night sleeping under ITN (56.7%), support providers particularly for mending/stitching holes/hanging ITNs (47.8%), role models for precautions related to washing ITN (39.3%), monitor for watching whether ITNs are properly used (32.0%), and norm setters for all-season sleeping under ITN (22.3%). Overall, summing the above concepts, the community’s perceived relevance of engaging students as change agents reached 43.7%.

**Table 2:** Community perspectives on students engaged as malaria education and change agents among households with school-going children, rural settings of Jimma zone, Ethiopia (n = 451)

Category of views: students were	Elaborative malaria message or action in terms of ITN	Standardized Mean ± SD
Credible sources of malaria messages	Students are knowledgeable, and working closely with the HEWs on malaria	64.30 ± 8.73
Reminders of messages and actions	Every family member should sleep under ITN every night	56.70 ± 7.45
Support providers whenever needed	Mending holes or hanging ITNs on beds	47.80 ± 5.60
Role model for actions	For washing ITN regularly (3 months)	39.33 ± 3.33
Monitor for actions	Following whether or not ITNs were utilized properly and as intended	32.00 ± 2.50
Norm setter for actions	Students were mostly observed while sleeping under ITNs at any season	22.33 ± 1.33
Overall engagement as educator	The summed pool of the above concepts	43.74 ± 4.26

HEWs: Health Extension Workers i.e. community based health workers

### Community recalls of school-based malaria and ITN messages

Table 3 presents the details of the community’s exposure to peer-learning assisted student malaria education. The study found a significant increase in the community’s reach with malaria messages through students by 44.5% (p < 0.001), based on 6 recent months’ recall. The content intensity of malaria messages to which respondents were exposed increased by 19.3%, p < 0.001. Sleeping under ITN appeared to be the top transmitted message content, at the endline (76.2%). This content was the second rank at baseline. Moreover, the messages about giving priority for the most vulnerable segments (pregnant women and children of under-5 years old) and careful washing of ITN significantly changed over the intervention periods by 28.8 and 30.9% (p < 0.001), respectively. There was an overtime increase in source repetition by 40.6% among HHs exposed to malaria messages by students. Community health workers and radio were the two main other sources of malaria messages among HHs that were exposed to student messages. Additionally, a significant increase was observed on exposure to health workers (ES = 5.1%, p = 0.02).

**Table 3:** Changes in community exposure to malaria messages via peer learning assisted student led education, rural settings of Jimma Zone, 2017–2019 (Baseline, n = 762; End-line, n = 759)

Exposure variables	Students outreach to community overtime			Statistical tests	
	Baseline	End-line	Effect size (ES)		
	No (%)	No (%)	% (95% CI)	t/x^2^	p-value
Exposed to message by students	71 (13.3)	266 (57.8)	+ 44.5 (34.7,51.3)	218.1	< 0.001

Main message contents	#n = 568	#n = 2128			
Sleeping under ITN-every night	52 (73.2)	215 (76.2)	+3.0 (− 1.5, +8.2)	0.28	0.598
Environmental sanitation	55 (77.5)	174 (61.7)	− 15.8 (− 20.2, + 9.8)	6.18	0.013
Proper use of anti-malarial drugs	3 (4.2)	121 (42.9)	+ 38.7(51.4, 23.5)	32.25	< 0.001
Seek treatment for fever early	7 (9.9)	113 (40.1)	+ 30.2 (21.9, 39.3)	23.10	< 0.001
Priority to PWs &< 5 (ITN)	7 (9.9)	109 (38.7)	+ 28.8 (22.3, 34.6)	21.32	< 0.001
How to wash ITN	4 (5.6)	103 (36.5)	+ 30.9 (25.7, 37.8)	25.62	< 0.001
Indoor residual spraying (IRS)	1 (1.4)	91 (32.3)	+ 30.9 (21.5, 41.8)	28.03	< 0.001
Others*	13 (18.3)	16 (5.7)	− 12.6 (− 15.6, − 8.6)	12.01	< 0.001
Content intensity (overall count)	142 (25.0)	942 (44.3)	+ 19.3(14.3, 25.2)	5.93	< 0.001

Sources other than students	#n = 284	#n = 1064			
Health workers	57 (80.3)	486 (85.4)	+ 5.1 (2.4, 10.2)	11.46	0.02
Media**	27 (38.0)	197 (34.6)	− 3.4(− 9.8, + 10.0)	9.51	0.387
Community groups/networks	6 (8.5)	77 (13.5)	+5.0 (-8.2, +3.2)	2.61	0.863
Others***	9 (12.7)	43 (9.0)	− 3.7(− 2.4, − 1.5)	11.0	0.02
Repetition of source (overall count).	99 (34.9)	803 (75.5)	+ 40.6(32.7, 49.2)	11.46	0.03

ES: Effect Size; PW; Pregnant Women; ITN: Insecticide Treated Nets

*others: misconceptions about causes of malaria; ITN with torn don’t protect from malaria, etc

^**^Media: radio and TV;

***others: religious leaders at mosques, church, and community gatherings; print materials; market places; traditional associations, etc

# counts of person-to- all specific message content (for content intensity) or sources (repetition)

### Effectiveness of school-based community malaria education

Table 4 presents the details of changes in the community’s knowledge, perceptions and practices overtimes based on coverage and content intensity of students’ education.

**Table 4:** Changes in knowledge, perceptions, practices about malaria versus school-based exposure, rural settings of Jimma zone, Ethiopia, 2017–2019 (Baseline, n = 762; End-line, n = 759)

Key variables	Change over intervention periods			Statistical tests	Students’ education coverage and messages	
	Baseline	End-line	Effect size (ES)		Exposure status	Contents intensity
	No (%)	No (%)	% (95% CI)	t/x^2^(p-value)	Odds ratio-OR (95% CI)	ß coefficient (95%CI)
Knowledge						
Comprehensive knowledge	185 (24.3)	297 (39.1)	+14.8 (7.8, 20.1)	38.54 (< 0.001)	1.39 (0.74, 2.6)	1.82 (1.41, 2.62)*
Critical signs: feeling cold	588 (77.3)	573 (75.6)	− 1.7 (− 2.3, +2.9)	0.59 (0.442)	1.14 (0.50, 2.57)	3.71 (− 11.25, 18.66)
Critical signs: fever	456 (59.9)	567 (74.9)	+15.0 (12.3, 18.8)	38.75 (< 0.001)	1.10(0.52, 2.28)	4.63 (2.79,11.43)*
Critical signs: headache	480 (63.1)	514 (67.8)	+3.9 (− 1.5, +6.6)	3.77 (0.052)	1.06(0.54, 2.07)	7.62 (− 3.90,19.15)
Cause of malaria: mosquito bites	404 (53.2)	560 (74.0)	+20.8 (15.6, 26.8)	70.96 (< 0.001)	0.93 (0.47, 1.82)	6.14 (− 4.96,17.79)
Causes: misconceptions	663 (82.5)	338 (44.6)	− 37.9 (− 44.9,− 31.0)	− 10.75 (< 0.001)	1.41 (0.80, 2.48)	− 11.46 (− 19.94,− 2.99)*
Most at risk: pregnant women	582 (76.7)	586 (77.4)	+0.7 (− 0.6, +1.4)	0.16 (0.735)	0.89 (0.47, 1.71)	− 4.16 (− 14.97, 6.64)
Most at risk: < 5 years old children	672 (88.5)	564 (74.4)	− 13.9 (− 18.3, − 6.7)	50.18 (0.006)	0.87(0.45, 1.69)	5.54 (− 17.76,4.17)
ITN protect from malaria	429 (56.6)	597(78.8)	+22.2 (17.0, 27.1)	85.11(< 0.001)	1.93 (1.02, 3.76)*	3.32 (− 8.80,15.44)
Knowledge of how to care ITN	–	359 (47.5)	–	–	1.23 (1.12, 1.41)*	24.79 (5.01, 49.12)*
Fill puddles/stagnant water	625 (82.5)	342 (45.2)	− 37.3 (− 43.2, − 31.0)	228.0 (< 0.001)	0.77 (0.41,1.43)	− 3.41 (− 14.27,7.45)
Perceptions and attitude						
Perceived Threat (PT)	513 (67.3)	494 (65.1)	− 2.2 (− 2.8, − 1.7)	6.72 (0.012)	0.98 (0.97, 0.99)*	− 0.19 (− 0,38,− 0.01)
Attitude (overall control measures)	725(95.2)	694 (91.4)	− 3.8 (− 6.0, − 1.6)	− 9.34 (0.004)	0.97 (0.96, 0.98)*	− 0.17 (− 0.32,− 0.02)*
Attitude towards (ITN)	406 (53.3)	592 (78.2)	24.9(23.4,26.5)	34.04 (< 0.001)	1.04 (1.03, 1.05)*	0.20 (0.07,0.34)*
Self-Efficacy (overall measures)	556 (72.9)	617 (81.3)	+8.4 (8.3, 8.5)	7.97 (< 0.001)	1.01 (1.004, 1.02)*	0.02 (− 0.11,0.15)
Perceived efficacy (overall)	643 (84.0)	655 (86.3)	+2.3 (1.7, 3.3)	10.31 (< 0.001)	1.00 (0.99, 1.01)	− 0.08 (− 0,25,1.00)
Perceived efficacy (ITN)	480 (63.0)	606 (79.8)	+16.8 (15.3, 18.2)	23.00 (< 0.001)	1.04 (1.02, 1.05)*	0.15(− 0.02,0.32)
Message acceptance (overall)	564 (74.2)	640 (84.2)	+10.0 (4.0, 16.0)	8.79 (0.01)	1.10 (0.95,1.30)	7.52 (4.12, 12.70)*
ITN message acceptance	450 (59.0)	655 (86.3)	+27.3(25.1,29.5)	24.52 (< 0.001)	1.02(1.016, 1.03)*	8.08 (2.02,14.15)*
Practices						
People who slept under ITN	1497 (37.2)	2369 (63.0)	+25.8 (23.8,27.9)	23.53 (< 0.001)	1.85 (1.40, 2.42)*	8.16 (2.61, 13.70)*
< 5 years children slept under ITN^#^	340 (53.9)	410 (70.2)	+16.3 (4.8, 29.7)	9.34 (0.01)	2.00(0.13, 31.98)	28.57(− 26.68,83.82)
Pregnant women slept under ITN^##^	12 (46.2)	22 (71.0)	+24.8 (7.3, 43.2)	4.21 (0.01)	2.98 (1.14,7.84)*	6.80 (− 12.98,56.14)
Treatment seeking for fever **	70 (67.3)	518 (3.6)	+16.3 (11.4, 22.7)	5.23 (0.02)	1.05 (0.41,2.70)	1.62 (− 11,81,15.05)
Early treatment seeking for fever	18 (25.7)	21 (41.2)	+15.5 (5.3, 24.6)	3.22 (0.04)	1.41 (0.51,3.88)	− 5.36 (− 20.52,9.81)
Used any drug for fever	74 (71.2)	52 (85.2)	+14.1 (7.8, 21.1)	4.23 (0.03)	1.53 (0.55, 4.22)	− 2.98 (− 11.79,11.84)
Anti− malarial drug adherence***	4 (28.6)	5 (50.0)	+21.4 (− 12.5, +54.7)	3.12 (0.273)	0.43 (0.16,1.29)	− 0.79 (− 17.61,16.04)
Cleaning: Surrounding^###^	–	80 (10.5)	–	–	0.72 (0.41,1.33)	2.57 (− 8.40, 13.55)
Cleaning: Compound^####^	–	127 (16.7)	–	–	1.12 (0.63,1.82)	− 1.50 (− 10.66,7.66)
HH’s safe handling of IRS ****	4 (10.5)	79 (71.8)	+61.3 (44.0, 71.1)	53.56 (< 0.001)	3.94 (1.63,9.53)*	6.67 (− 9.50, 22.84)

*Statistical significance

**Prevalence of febrile illness (n = 165; baseline = 104(2.6%) and endline, n = 61(1.6%)

*** people who took anti-malarial drug(baseline, n = 14 and endline, n = 10)

****No plastering/painting done within 6 months of spray among HHs recently sprayed and painted (n = 148; baseline = 38 and endline = 110),

^#^Under-5 years old children (baseline = 631 and endline, n = 584),

^##^ Pregnant women (baseline, n = 26 and endline, n = 31),

^###^HHs engaged in cleaning surrounding at least once per month

^####^HHs engaged in cleaning their compound from anything suitable for mosquito breeding at least once per week

#### Effects on knowledge and perceptions of malaria and ITN

Over the intervention periods, there were positive significant changes in comprehensive knowledge about malaria (ES = 14.8%, p < 0.001), identifying fever as one of its critical signs (ES = 15.0%, p < 0.001), mosquito bite as its correct cause (ES = 20.8, p < 0.001), mentioning ITN as one of the effective protective methods (ES = 22.2%, p < 0.001), attitude towards ITN (ES = 24.9%, p < 0.001), perceived efficacy of using ITN (ES = 23.0, p < 0.001), and evaluating and accepting ITN as an important tool for controlling the danger of malaria (ES = 27.3%, p < 0.001). There was a significant improvement pertaining misconceptions about causes of malaria, i.e., empty stomach, cold weather, and eating dirty foods (ES = − 37.9%, p < 0.001). Nonetheless, perceived threat from malaria (ES = − 2.2%, p = 0.012), and overall attitudes of malaria control measures (ES = − 3.8, p = 0.004) were negatively correlated with exposure to school-based community education.

As noted from the columns about effects of students’ education in Table 4, exposure status or content intensity of malaria education by students significantly improved comprehensive knowledge about malaria (β = +1.82), identifying fever as its critical sign (β = 4.63), knowledge of caring for ITN, i.e., washing and drying, and mending/stitching for re-use (β = 24.79), identifying ITN as one of the effective preventive methods (OR = 1.93), attitude towards ITN (β = 0.20), and perceived efficacy of ITN (OR = 1.04). Most of the changes were related to the content intensity of the education rather than mere exposure, i.e., for one unit increase in the content intensity of messages there were average increases in values of β respective to each variable. These indicate the need to emphasize contents of the messages that were taken to parents or neighbors about malaria by students. The observed negative changes reported earlier in terms of perceived threat, overall attitude, misconceptions about causes were also related to exposure or content.

#### Effect on the practice of ITN utilization

Table 4 shows that there was a significant change in practices about malaria over intervention periods and due to exposure to or content of education delivered by students. There was positive significant changes overtime regarding ITN usage by people in families (ES = 25.8%, p < 0.001). The utilization of ITN was significantly associated with exposure to or content intensity of the students’ educating their community. Of people who slept in the surveyed households, those from families exposed to students’ education were averagely 1.85 times more likely to sleep under ITN the previous night of the survey compared to those who were not exposed. Additionally, people who slept under ITN the night previous to the survey were having an 8.16 average excess score of content intensity compared to those who did not sleep under ITN. The act of giving priority for pregnant women so that they sleep under ITN was improved by 2.98 times among families exposed to messages delivered by students compared to those who were not exposed.

### Changes in predictors of ITN over the intervention periods

The above reports indicated that ITN was the dominant practice change observed following the students’ teaching their parents at home. Consequently, the patterns of change in the practice of ITN and the predictors were further demonstrated based on socio-demographic factors, access to ITN, exposure to education, and parental perceived engagement of students (Table 5). Only variables with significant effects are presented in the Table.

**Table 5:** Changes in pattern and predictors of ITN utilization, school− based education targeted rural settings of Jimma Zone, Ethiopia (Baseline, n = 762 and End− line, n = 759)

Predictors	ITN utilization practice			
	Baseline	End-line	Ratio of change	Percent of change
	OR (95% CI)	OR (95% CI)	Change ratio	ES % (95%CI)
Intervention				
End-line	1	2.87 (2.62,3.15)	2.87 (2.62,3.15)	+ 187.0 (162.0–215)
Districts				
Nono-Benja	0.42(0.34,0.52)	0.70 (0.56,0.88)	1.67 (1.65,1.69)	+ 67.0 (65.0,69.0)
Limmu-Kosa	0.39 (0.31,0.50)	0.49 (0.39,0.63)	1.25 (1.23,1.26)	+ 25.6 (22.6,26.0)
Gera	0.28 (0.23,0.36)	1.03 (0.83,1.29)	3.58 (2.96,3.68)	+ 258 (196,268)
Shebe-Sombo	1.36 (1.13,1.64)	1.85 (1.50,2.30)	1.36 (1.33,1.40)	+ 36.0 (32.7,40.2)
Botor-Tolay	1	1	1	Reference
Sex of family members				
Male	1.24 (1.10,1.41)	1.31 (1.14,1.49)	1.06 (1.04,1.07)	+ 6.0 (4.0,7.0)
Ethnic groups				
Oromo	1	1	1	Reference
Amhara	1.77 (1.19,2.61)	1.19 (0.80,1.77)	0.68 (0.67,0.91)	− 32.0 (− 33.0,− 9)
Others	2.67 (0.83,7.44)	7.16 (0.96,42.3)	2.68 (1.16, 5.69)	+168 (16.0469)
Ownership and access to ITN				
Own functional ITN				
Yes	4.40 (3.66,5.89)	1.77 (1.43,2.45)	0.40 (0.39,0.42)	− 59.8 (− 61.0,− 58.4)
Number of ITN	2.15 (1.84,2.51)	1.61 (1.39,1.86)	0.75 (0.74,0.760	− 25.1 (− 26.0,− 24.5)
Access (1 ITN for 2 people)	16.7 (9.8,28.5)	10.0 (5.8,17.4)	0.61 (0.59,0.62)	− 39.4 (− 40.8,− 0.38)
ITN with hole	0.74 (0.56,0.93)	1.04 (0.86,1.26)	1.41 (1.36, 1.54)	+ 40.5 (35.5, 53.6)
Number of ITN saved	0.21 (0.16,0.28)	0.33 (0.26,0.42)	1.57 (1.50,1.63)	+ 43.0 (37.0,50.0)
Exposure-to school based education				
Content intensity	0.99 (0.96,1.03)	1.02 (1.01,1.04)	1.03 (1.01,1.05)	+ 3.0 (1.0,5.0)
Comprehensive knowledge	1.89 (1.35, 2.64)	1.17 (0.86, 1.60)	0.62(0.61,0.64)	− 38.1 (− 39.4,− 36.3)
Cause (mosquito bite)				
Yes	2.08 (1.54,2.80)	1.26 (0.90,1.76)	0.61 (0.58, 0.63)	− 39.4 (− 41.6,− 37.1)
Attitude (ITN)	1.10 (1.03,1.18)	1.06 (1.01,1.10)	0.96 (0.93, 0.98)	− 3.6 (− 6.8, − 1.9)
Perceived efficacy	1.02 (1.01,1.03)	1.03 (1.02,1.04)	1.01 (1.00,1.01)	+ 1.0 (0.9,1.1)
Acceptance of ITN				
For danger control	1.01 (1.004,1.02)	1.02 (1.01, 1.03)	1.02 (1.01,1.03)	+ 2.0 (0.6,2.8)
Community perceived engagement of students as				
Good reminders	–	1.53 (1.11,2.11)	–	

OR: odds ratio; ES: effect size

#### Socio-demographic predictors of ITN utilization

Table 5 presents the details of changes in predictors of ITN utilization overtimes. The utilization of ITN by people who slept in a household averagely increased by 2.87 at the endline compared to baseline, i.e., 187% excess percentage of increase. The study revealed that a spatial variation predicted utilization of ITNs. For example, over the intervention periods, people living in Limmu-Kossa and Nono-Benja districts remained less users of ITN compared to Botor-Tolay, while people who mostly used ITN lived in Shebe-Sombo district. There were significant positive modifications in odds ratios across all the districts compared to Botor-Tolay; the highest change in probability of using ITN occurred among people living in Gera and Nono-Benja with a respective change of 258% and 67%. Overtime, males as household member became more users of ITN compared to females, with 6% average change in odds ratios. Changes in odds ratios of ITN use based on ethnic groups resulted in no significant difference at endline, which was present at baseline.

#### Ownership and access predictors of ITN utilization

As presented in Table 5, even though the student-led education was not accompanied by the provision of resources such as ITNs, they worked closely with local health offices to resolve access challenges. In this study ownership of ITN (baseline, OR = 4.40, and endline, OR = 1.77), number of ITNs owned (baseline, OR = 2.15 and endline, OR = 1.61), and access to one ITN for every 2 people in households (baseline, OR = 16.7 and endline, OR = 10.0) remained predictors of ITN utilization over intervention periods. Access remains a stronger predictor, i.e., the probability of using ITN was 10-fold more among people accessed with ITN at endline, which was 16.7 times at baseline. The above figures show that the magnitude of effects of ownership, number and access of ITN significantly reduced at endline compared to baseline, with percentages of change by − 59.8, − 25.1, and − 39.4%, respectively. These figures indicate the challenges of ITN availability and access that were resolved at endline compared to baseline. Interestingly, the ITNs observed to have holes negatively predicted the use of ITN (OR = 0.74, 95% CI: 0.54, 0.93) at baseline, while at endline people mended the holes and improved the usability of the ITNs, i.e., there was no difference in using ITN because it had a hole (OR = 1.04, 95% CI: 0.86, 1.26). The percentage change of odds of improving ITN for usability was 40.5%. Additionally, the odds ratios of the number of ITNs saved as a negative predictor was increased by 43.0% at endline compared to baseline, potentially indicating misdistribution practices of ITNs that increased overtime at reducing effect on its use.

#### School-based education and perceptions related predictors of ITN utilization

As presented in Table 5, the study revealed that the content intensity of school-based education became one of the predictors of ITN at endline, which was not a predictor at baseline. The percentage change in odds ratio over the intervention periods was only 3.0%, indicating the need to advance the role of schools in prediction of ITN utilization. Perceptions (attitudes and perceived efficacy of ITN) and evaluative acceptance of ITN as a relevant tool for controlling the danger of malaria remained positive predictors of ITN along the intervention periods.

## Discussion

The current study investigated the effectiveness of engaging primary school (grade 5–8) students in teaching their parents at home in rural settings by following objective-based and flipchart-supported peer discussion. From the post-intervention survey, students were perceived by parents to be credible messengers (64.3%) and good reminders (56.7%). The study generated significant changes in exposure, knowledge, perceptions, and practices over the intervention periods (2017–2019). There was a significant positive change in exposure to malaria messages delivered by students in terms of reach (ES = 44.5%) and content intensity (ES = 19.3%). 

Regression analysis of perceptions and practices based on exposure status and content intensity of messages delivered by students resulted in improvements in comprehensive knowledge, misconceptions about causes of malaria, ITN related perceptions (attitude, self-efficacy, perceived efficacy, and how to care for it), and ITN utilization. Previous school-based studies in Ghana, Lao PDR, Kenya, and Thailand reported similar findings: reductions in misconceptions, and improvement in practices both among students and community adults [26–28, 31]. Significant negative changes were observed of the perceived threat and overall attitude towards control measures. Interestingly, most of the changes were correlated with the content intensity of messages transmitted rather than reach. This indicates that emphasis should be given to carefully identified content of education while adopting a school-based education strategy for malaria control measures.

The magnitude over intervention periods and amounts of observed change needs some discussion regarding some variables. For example, in this study the amount of change in comprehensive knowledge about malaria was +14.5%. However, even post-intervention knowledge (39.3%) was very low compared to amounts reported in studies in Africa [49–51] and the national elimination plans, i.e., near 100% for specific item: knowledge of mosquito bite as a correct cause of malaria [12, 13]. Studies [49, 50] measured specific knowledge items unlike the current study that comprehensively measured knowledge. One study in Equatorial Guinea reported closer value, 35%, of combined knowledge score on malaria among rural sub-groups, which is comparable in terms of study population and measurement [52]. The magnitude of ITN utilization is another variable needing explanation. The size of changes observed over intervention was as high as + 25.8%. Similarly, ITN messages were accepted in favor of controlling the danger of malaria with nearly equivalent similar change size, ES = 27.3%. Theoretically (according to EPPM), if messages are positively accepted in favour of danger control, one can expect the related behavioural uptake, ITN use in this case [47]. Even the endline magnitude, 63%, of ITN use was found to be low. According to the NSMP III plans, at least 80% of people at risk would use ITN [12].

Negative but slight changes were observed over time regarding perceived threat (ES = − 2.2%, p = 0.012) and overall attitude towards malaria control measures (ES = − 3.8%, p = 0.004) and that was also negatively correlated with exposure to and content of messages delivered by students. This looks strange, particularly compared to positive changes in attitude towards ITN over time and with school-based messages. Perhaps, students were unintentionally promoting the outcome of preventive actions with particular emphasis on ITN with less attention paid to perceived risk and severity of malaria. Pieces of evidence report malaria incidence constantly declining throughout the globe, including in Jimma, and so too risk perception and attitudes about treatment services [7–13, 21, 41, 53–55]. The finding suggests that people were more concerned about ITN than other control measures.

The finding from this study indicates that the effect of school-based education was evident regarding ITN utilization by pregnant women and people who slept in HHs the night before the surveys. There was nearly a two-fold (OR = 1.85) ITN usage among HHs exposed to students compared to those not exposed. Similar studies on school-based education in Africa reported effective changes in ITN usage [29, 31]. ITN utilization among people in HHs with priority to pregnant women and < 5 years old children is considered a frontline method of prevention according to RBM-SBCC indicators [19–21]. The prevalence of ITN use observed at endline was higher than one-time cross-sectional studies that were conducted in Ethiopia [36, 37]. In this study, improvement in ITN utilization might have caused a reduced perceived threat or attitude towards other malaria control measures due to feeling protected by ITN. Conversely, the reductions in threat and overall attitude could have a long-term and negative effect on ITN utilization. The relationship between the two needs further investigation.

This study assessed predictors of ITN utilization and their patterns of change over the intervention periods. Spatial variations (study districts), gender, ownership-access, exposure to content of school-based messages, perceptions (attitude, perceived efficacy) towards ITN, and a parental perception that students were good reminders were main predictors of ITN utilization. Studies conducted in different regions or settings in Ethiopia reported differences in the prevalence of ITN utilization ranging from 37 to 91% [36, 37, 51]. Access has been a challenge to ITN usage [37–41]. Many studies reported that attitudes predict ITN use [36, 49, 52, 54]. Interestingly, the number of ITNs that were saved for future is losing the extent to which it predicted ITN utilization (OR changed from 0.21 to 0.33), indicating that the saved ITN did not necessarly imply that people are not using them; rather the saving may indicate excess presence of the ITNs or sense of security.

Over the intervention periods, the magnitude of effects on ITN utilization that were due to access and spatial variations were significantly reduced as percentage changes in odds ratios. Perhaps, this is because of school-based education coverage and improved access to ITN across the study districts [38, 39, 41]. Mass distribution programmes may be the reason for access [7–12, 19]. On the contrary, there was a minor increase in the percentage of effect on ITN usage due to exposure to the content of school-based messages, perceived efficacy, and ITN-related message acceptance. This indicates the need to strengthen school-based education intervention towards malaria control.

### Strength and limitations

There are few studies focused on demonstrating that teaching students in school increases perceptions and utilization of ITN at home that the current study addressed. The current study reported changes in ITN usage overtime based on exposure to messages delivered by students. The study was conducted based on behaviour change theories, and RBM/SBCC indicators. Nonetheless, there was no control group for the study.

## Conclusions

Post-exposure to school-based community education, there was a promising percentage of parents who perceived that students are credible messengers and good reminders of every-night use of ITN by family members. Peer-learning assisted primary school students can improve community exposure to malaria and ITN messages. The content intensity of messages delivered by students improves households’ knowledge about malaria prevention, misconceptions,, attitude toward and utilization of ITN. The reductions in the perceived threat from malaria and an overall attitude toward control measures other than ITN were associated to the school-based education. This could be reported as an unintended effect and area of emphasis in future communication on malaria. The current level of reduction in threat and overall attitude does not affect ITN utilization although a potential long-term effect is not estimated. The spatial difference, access to ITN, exposure to messages delivered by students, perceived efficacy, and perceived engagement of students in malaria education as good reminders were predictors of ITN utilization. The pattern of changes shows challenges related to access and spatial variations are reducing. The effect of exposure to messages from students on ITN usage showed a slight increment and scalable. Even though attitude towards ITN positively predicted ITN use over time, the percentage of effect is reducing. This suggests the need to set ITN use a social norm before it results in a fall of the usage. The content intensity of messages delivered by students is far better than mere exposure status to initiate perceptions and ITN utilization behaviour changes. A community education supported by students’ peer discussion reinforces behaviour change to be more positive towards malaria control efforts. A school-based education strategy is recommendable for malaria combating efforts.

## Data Availability

All data generated or analysed during this study are included in this published article.

## Abbreviations

EMIS: Ethiopian Malaria Indicator Survey
ES: Effect Size
GMP: Global Malaria Programmes
GTS: Global Technical Strategy
HHs: Households
ITNs: Insecticide Treated Nets
MEF: Malaria Elimination Framework
NMSP: National Malaria Strategic Plans
RBM: Roll Back Malaria
SBCC: Social and Behaviour Change Communication
WHO: World Health Organization

## References

[1] WHO (2016). Training module on malaria elimination: malaria elimination guide for participants.

[2] WHO (2015). Global technical strategy for malaria 2016–2030.

[3] Roll Back Malaria (2000–25) (2000). Framework for monitoring & progress evaluating outcomes, and impact.

[4] Roll Back Malaria, MEASURE Evaluation, World Health Organization, UNICEF (2004). Guidelines for core population coverage indicators for Roll Back Malaria: to be obtained from Household Surveys.

[5] Roll Back Malaria, MEASURE Evaluation, USAID, UNICEF, USAID, World Health Organization, MACEPA, CDC (2009). Guidelines for core population-based indicators.

[6] USAID. President’s Malaria Initiative Strategy (2015–2020). U.S. Agency for International Development, 1300 Pennsylvania Avenue, NW, Washington, DC 20523. 2015.

[7] Federal Ministry of Health of Ethiopia, PATH Malaria Control, and Elimination Partnership in Africa (MACEPA). Ethiopia: Accelerating Toward Malaria Elimination: Stakeholder Perspectives. Addis Ababa, 2015.

[8] Federal Ministry of Health of Ethiopia. National Strategic Plan for Malaria Prevention Control and Elimination in Ethiopia–2006–2010. Addis Ababa, 2010.

[9] Federal Ministry of Health of Ethiopia (2005). Health Sector Strategic Plan (HSDP-III) 2005/6-2009/10.

[10] Federal Ministry of Health of Ethiopia. National Strategic Plan for Malaria Prevention Control and Elimination in Ethiopia–2011–2015. Addis Ababa, 2010.

[11] Federal Ministry of Health of Ethiopia (2010). Health Sector Strategic Plan (HSDP-IV) 2010/11-2014/15.

[12] Federal Ministry of Health of Ethiopia. National Strategic Plan for Malaria Prevention Control and Elimination in Ethiopia, 2015–2020. Addis Ababa, 2014.

[13] Federal Ministry of Health of Ethiopia. Health Sector Transformation Plan (HSTP) 2015/16-2019/20. Addis Ababa, 2015.

[14] Federal Ministry of Health of Ethiopia (2017). PATH malaria control and elimination partnership in Africa (MACEPA).

[15] WHO (2017). Global Malaria Programme: a framework for malaria elimination.

[16] Federal Ministry of Health of Ethiopia (2016). Ethiopia National Malaria Indicator Survey 2015.

[17] U.S. Global Malaria Coordinator, U.S. President’s Malaria Initiative Ethiopia. Malaria Operational Plan FY, 2019.

[18] Evaluation Task Force of Roll Back Malaria’s Monitoring and Evaluation Reference Group (2019). Framework for Evaluating National Malaria Programs in Moderate and Low Transmission Settings.

[19] Roll Back Malaria Partnership to End Malaria (2018). The Strategic Framework for Malaria Social and Behavior Change Communication 2018-2030.

[20] RBM Partnership to End Malaria (2017). Malaria social and behavior change communication indicator reference guide.

[21] RBM/MERG. Household survey indicators for malaria control. MEASURE Evaluation, The Demographic and Health Surveys Program, President’s Malaria Initiative, Roll Back Malaria Partnership, United Nations Children’s Fund, World Health Organization. Survey and Indicator Task Force of the Roll Back Malaria Monitoring & Evaluation Reference Group; 2018.

[22] Breakthrough ACTION Guyana. Using Human-Centered Design to Improve Malaria Outcomes in Regions 7 and 8 in Guyana, 2017.

[23] Clarke SE, Jukes MCH, Njagi JK, Khasakhala L, Cundill B, Otido J (2008). Effect of intermittent preventive treatment of malaria on health and education in school children: a cluster-randomized, double-blind, placebo-controlled trial. Lancet.

[24] Add Logos. School Health Program: Malaria Awareness And Action, Manual for Malaria Education Program Development, and Implementation. Stop Malaria Uganda. 2014.

[25] Price OA. School-centered approaches to improve community health: lessons from school-based health centers. Economic Studies at Brookings. 2016;5.

[26] Nonaka D, Kobayashi J, Jimba M, Vilaysouk B, Tsukamoto K, Kano S (2008). Malaria education from school to the community in Oudomxay province, Lao PDR. Parasitol Int.

[27] Onyango-Ouma W, Aagaard-Hansen J, Jensen BB (2005). The potential of schoolchildren as health change agents in rural western Kenya. Soc Sci Med.

[28] Okabayashi H, Thongthein P, Singhasvanon P, Waikagul J, Looareesuwan S, Jimba M (2006). Keys to success for a school-based malaria control program in primary schools in Thailand. Parasitol Int.

[29] Owusu-Addo E, Owusu-Addo SB (2014). Effectiveness of health education in community-based malaria prevention and control interventions in sub-Saharan Africa: a systematic review. J Biol Agric Healthc..

[30] Nankabirwa J, Brooker SJ, Clarke SE, Fernando D, Gitonga CW, Schellenberg D (2014). Malaria in school-age children in Africa: an increasingly important challenge. Trop Med Int Health..

[31] Ayi I, Nonaka D, Adjovu JK, Hanafusa S, Jimba M, Bosompem KM (2010). School-based participatory health education for malaria control in Ghana: engaging children as health messengers. Malar J..

[32] Umwangange ML, Chironda G, Mukeshimana M (2018). Knowledge, attitude, and practice towards malaria prevention among school children aged 5–14 years in sub-Saharan Africa: a review of literature. Rwanda J Med Health Sci..

[33] Masaninga F, Mukumbuta N, Ndhlovu K, Hamainza B, Wamulume P, Chanda E (2018). Insecticide treated nets mass distribution campaign: benefits and lessons in Zambia. Malar J..

[34] Keating J, Hutchinson P, Miller JM, Bennett A, Larsen D, Hamaninza B (2012). A quasi-experimental evaluation of an interpersonal communication intervention to increase insecticide-treated net use among children in Zambia. Malar J..

[35] The Health Communication Capacity Collaborative (HC3) (2017). Malaria SBCC Evidence Literature Review.

[36] Admasie A, Zemba A, Paulos W (2018). Insecticide-treated nets utilization and associated factors among under-5 years old children in Mirab-Abaya District, Gamo-Gofa Zone, Ethiopia. Front Public Health..

[37] Watiro AH, Awoke W (2016). Insecticide-treated net ownership and utilization and factors that influence their use in Itang, Gambella region, Ethiopia: a cross-sectional study. Risk Manag Healthc Policy..

[38] Seyoum D, Speybroeck N, Duchateau L, Brandt P, Rosas-Aguirre A (2017). Long-lasting insecticide net ownership, access, and use in Southwest Ethiopia: a community-based cross-sectional study. Int J Environ Res Public Health..

[39] Birhanu Z, Abebe L, Sudhakar M, Dissanayake G, Yihdego Y, Alemayehu G (2015). Access to and use gaps of insecticide-treated nets among communities in Jimma Zone, southwestern Ethiopia: baseline results from malaria education interventions. BMC Public Health..

[40] Kilian A, Koenker H, Paintain L (2013). Estimating population access to insecticide-treated nets from administrative data: correction factor is needed. Malar J..

[41] Taffese HS, Hemming-Schroeder E, Koepfli C, Tesfaye G, Lee M, Kazura J (2018). Malaria epidemiology and interventions in Ethiopia from 2001 to 2016. Infect Dis Poverty..

[42] World Health Organization (2017). Global school health initiatives: achieving health and education outcomes: Report of a meeting, Bangkok, Thailand, 23–25 November 2015.

[43] Federal Ministry of Education of Ethiopia (2012). National School Health And Nutrition Strategy.

[44] Simon B. Malaria control in schools: A toolkit on effective education sector responses to malaria in Africa. Partnership for Child Development, London School of Hygiene and Tropical Medicine, Kenya Medical Research Institute-Wellcome Trust Research Programme, World Bank, 2009.

[45] PMI/VectorWorks Ghana. Promoting malaria prevention through primary schools: Communication Guide for Teachers. President’s Malaria Initiative, Ghana Health Services, Ghana Education services, John Hopkins Center for Communication programs. 2016.

[46] Kebede Y, Abebe L, Alemayehu G, Sudhakar M, Birhanu Z (2020). School-based social and behavior change communication (SBCC) advances community exposure to malaria messages, acceptance, and preventive practices in Ethiopia: a pre-posttest study. PLoS ONE.

[47] Witte K (1994). Fear control and danger control a test of the extended parallel process model. Commun Monogr..

[48] Wikipedia, the free encyclopedia. Diffusion of innovations. https://en.wikipedia.org/wiki/Diffusion_of_innovations.

[49] Shimaponda-Mataa NM, Tembo-Mwase E, Gebreslasie M, Mukaratirwa S (2017). Knowledge, attitudes, and practices in the control and prevention of malaria in four endemic provinces of Zambia. S Afr J Infect Dis..

[50] Cox SN, Guidera KE, Simon MJ, Sanny-Nonyane BA, Brieger W, Bornman MS (2018). Interactive malaria education intervention and its effect on community participant knowledge: the malaria awareness program in Vhembe District, Limpopo, South Africa. Int Q Community Health Educ..

[51] Alemu MB, Asnake MA, Lemma MY, Melak MF, Yenit MK (2018). Utilization of insecticide-treated bed net and associated factors among households of Kola Diba town, North Gondar, Amhara region. Ethiopia. BMC Res Notes..

[52] Romay-Barja M, Ncogo P, Nseng G, Santana-Morales MA, Herrador Z, Berzosa P (2016). Caregivers’ Malaria Knowledge, Beliefs and Attitudes, and Related Factors in the Bata District. Equatorial Guinea. PLoS ONE..

[53] Jemal A, Ketema T (2019). A declining pattern of malaria prevalence in Asendabo Health Center Jimma zone, Southwest Ethiopia. BMC Res Notes..

[54] Koenker HM, Loll D, Rweyemamu D, Ali AS (2013). A good night’s sleep and the habit of net use: perceptions of risk and reasons for bed net use in Bukoba and Zanzibar. Malar J..

[55] Bauch JA, Gu JJ, Msellem M, Martensson A, Ali AS, Gosling R (2013). Perception of malaria risk in a setting of reduced malaria transmission: a qualitative study in Zanzibar. Malar J..

